# Hepatic and Renal Toxicity Induced by TiO_2_ Nanoparticles in Rats: A Morphological and Metabonomic Study

**DOI:** 10.1155/2019/5767012

**Published:** 2019-03-03

**Authors:** Xavier Valentini, Pascaline Rugira, Annica Frau, Vanessa Tagliatti, Raphaël Conotte, Sophie Laurent, Jean-Marie Colet, Denis Nonclercq

**Affiliations:** ^1^Laboratory of Histology, University of Mons, Institute for Health Sciences and Technology, Faculty of Medicine and Pharmacy, 23 Place du Parc, 7000 Mons, Belgium; ^2^Laboratory of Human Biology & Toxicology, University of Mons, Institute for Health Sciences and Technology, Faculty of Medicine and Pharmacy, 23 Place du Parc, 7000 Mons, Belgium; ^3^Laboratory of General, Organic and Biomedical Chemistry, NMR and Molecular Imaging Laboratory, University of Mons, Institute for Health Sciences and Technology and Biosciences, Faculty of Medicine and Pharmacy, 23 Place du Parc, 7000 Mons, Belgium; ^4^Center for Microscopy and Molecular Imaging (CMMI), 6041 Gosselies, Belgium

## Abstract

Titanium dioxide (TiO_2_) nanoparticles (NPs) are produced abundantly and are frequently used as a white pigment in the manufacture of paints, foods, paper, and toothpaste. Despite the wide ranges of uses, there is a lack of information on the impact of NPs on animal and human health. In the present study, rats were exposed to different doses of TiO_2_ nanoparticles and sacrificed, respectively, 4 days, 1 month, and 2 months after treatment. Dosage of TiO_2_ in tissues was performed by ICP-AES and revealed an important accumulation of TiO_2_ in the liver. The nanoparticles induced morphological and physiological alterations in liver and kidney. In the liver, these alterations mainly affect the hepatocytes located around the centrilobular veins. These cells were the site of an oxidative stress evidenced by immunocytochemical detection of 4-hydroxynonenal (4-HNE). Kupffer cells are also the site of an important oxidative stress following the massive internalization of TiO_2_ nanoparticles. Enzymatic markers of liver and kidney functions (such as AST and uric acid) are also disrupted only in animals exposed to highest doses. The metabonomic approach allowed us to detect modifications in urine samples already detectable after 4 days in animals treated at the lowest dose. This metabonomic pattern testifies an oxidative stress as well as renal and hepatic alterations.

## 1. Introduction

Currently, the use of nanotechnology, including nanoparticles, has dramatically increased in different fields such as medicine, cosmetics, energy, chemical, and textile industries [1]. Nanomaterials vary in size from 1 to 100 nm [2]. Titanium dioxide nanoparticles (TiO_2_ NPs) are mostly used in a large panel of applications such as the manufacture of plastics, as adjuvant in pharmaceutical pills composition; they are also widely used as bleaching agents in the paper industry, in the production of paints, cosmetics, sunscreens, and toothpastes [3]. Since last decade, nearly 6 million tons of TiO_2_ was produced worldwide as a pigment [4] and the percentage of nano-TiO_2_ was close to 5% of the total production [5]. The same report suggested that, by 2023, up to 50% TiO_2_ might be manufactured in nanoform. Inside the panel of applications, these nanoparticles are used on a large scale as food additives [6]. Despite the wide range of applications and our daily exposure to these particles, there is a lack of information on animal and human health and on the impact on the environment. The human body can be exposed to these NPs through different routes of exposure such as inhalation, ingestion, dermal penetration, and injection [7].

Nowadays, it is impossible to deny that NPs are deleterious for the human health. For example, welders or coal miners succumbed to disease following exposure to nanoparticles of metallic oxides or silica [8]. Ferin et al. [9] were the first to describe that a nanosized material may elicit an adverse biological response. This study highlighted the size-dependent toxicity of TiO_2_ NPs leading to an acute inflammatory reaction in lungs compared to microparticles.* In vitro* and* in vivo* studies showed that nanoparticle-induced lung injury and pulmonary fibrosis may be linked to the reactive oxygen species (ROS) production [10]. ROS include superoxide anion radical (^∙^O_2_^−^), hydroxyl radicals (^*∙*^OH), and hydrogen peroxide (H_2_O_2_). ROS are constantly produced by normal cellular events, with a major source being aerobic respiration, but ROS produced during these events are generally counteracted by several antioxidant molecules [11]. Several studies have demonstrated that TiO_2_ NPs could induce oxidative stress by the generation of ROS, reduced glutathione (GSH) depletion, and reduction of mitochondrial membrane potential leading to cell death. Oxidative stress occurs when there is an imbalance between oxidant and antioxidant within the cell [12]. The activation of macrophage increases oxidation in the cell by generation of the superoxide anion which is converted into the hydroxyl radical (^*∙*^OH). The molecules that possess unpaired electron(s) are highly unstable. This study also indicates that cells performing phagocytosis of xenobiotics can produce ROS. The production of ROS and the generation of an oxidative stress are extremely deleterious for the cell causing a reduction of metabolism and an increase in the production of inflammatory mediators. It may be associated with illnesses such as asthma, cardiovascular diseases, and even tumour formation [13].

The large surface area of NPs increases their capacity to produce ROS such as hydrogen peroxide and hydroxyl radicals. ROS production leads both to cytotoxicity and to genotoxicity. The accumulation of ROS induces oxidative damage of macromolecules including lipid, protein, and DNA peroxidation. If the oxidative stress occurs in a cell, it can lead to alterations in signal transduction and gene expression for metabolism and mitogen pathways [14]. Liang et al. [15] showed that, after TiO_2_ NP exposure, the superoxide dismutase (SOD) activity in plasma and the glutathione peroxidase (GPx) activity in the kidneys were significantly decreased and the malondialdehyde (MDA) levels of the liver and kidneys were significantly increased. Pelclova et al. [16] demonstrated the presence of different markers of lipid peroxidation such as malondialdehyde (MDA), 4-hydroxyhexenal (HHE), and 4-hydroxynonenal (HNE) in the exhaled breath condensate and urine of workers exposed to air pollutants including TiO_2_ NPs.

Oxidative stress induced by many toxic compounds causes disruption of several major metabolic pathways within target organs such as the liver or kidneys [17]. These metabolic dysfunctions induce changes in the urinary metabolome. The metabonomic fingerprints are increasingly used as a means of early detection of toxicity even when the toxic agent is administered at very low doses [18]. However, the metabonomic approach has been rarely used to study the disturbance of cell metabolism induced by TiO_2_ nanoparticles. Only one* in vitro* study performed on mouse fibroblast cells showed a perturbation of amino acid synthesis pathways [19]. Another study performed in* Caenorhabditis elegans* demonstrated a modification of metabonomic signature induced by TiO_2_ NPs [20]. At present, no* in vivo* studies have been carried out on urinary metabonomic changes related to exposure to TiO_2_ nanoparticles in mammals or humans. On the other hand, the location of the cells most exposed to oxidative stress in the target organs such as the kidney and the liver is also still largely unknown as well as the histopathologic lesions induced by TiO_2_ nanoparticles.

In the present study, the aim was to determine the toxicity of TiO_2_ NPs in rats exposed to different doses and euthanized after different time intervals. The results presented in this paper pointed to an early disruption of the metabonomic signature in the urine of the animals exposed to the lowest doses while they showed no sign of morphological or ultrastructural alteration. On the other hand, in high doses treated rats, our results showed a correlation between the oxidant stress evidenced by immunocytochemistry and the disturbance of the mitochondrial activity demonstrated by metabonomics. These data obtained by multidisciplinary approach (ICP, Immunohistochemistry, TEM, and metabonomic) give an originality to our work to better understand the relationship between oxidative stress, metabolic alteration, and histological lesion induced by TiO_2_ NPs exposure.

## 2. Material and Methods

### 2.1. Nanoparticles Characterization

TiO_2_ nanoparticles [CAS N°: 1317.70.0] were purchased from Sigma-Aldrich®, UK. As specified by the supplier, TiO_2_ NPs used in this study were titanium (IV) oxide, anatase with a purity of 99.7%, based on trace of metal analysis. All suspensions were prepared in sterile phosphate buffer saline (PBS). In order to reduce size of nanoparticles aggregates, NPs were sonicated in probe sonicator (UP200S, dr.Hielscher Ultrasound technology (GmbH), 50/60Hz; 230V) for 3 runs of 30 min as detailed in previous publications [21, 22]. The shape and the average aggregate size of TiO_2_ NPs were evaluated by transmission electron microscopy using a Zeiss® LEO 906E Electron microscope at 80kV. The mean size of aggregates was evaluated on 20 TEM pictures picked at random covering more than 100 aggregates. The morphometric analysis of the pictures was performed by using the software Zeiss® KS400. The measurements of the size distribution were also performed by dynamic light scattering (DLS) method. DLS and zeta potential of the nanoparticles suspended in aqueous medium were carried out on a Zetasizer Nano ZS (Malvern Instruments®, UK) using laser He-Ne (633 nm). The zeta potential was determined directly in a saline solution (NaCl 0.01 mM) and the pH was adjusted by adding 0.1-0.001 mM HNO_3_ or NaOH solution.

### 2.2. Animals and Treatment

All experiments were performed on 2-month-old male Wistar rats weighing 200-250g [22, 23] originally obtained from Charles River (Belgium). Animals were treated according to the guideline specified by the Belgian Ministry of Trade and Agriculture and under the control of the UMons-ethical commission (agreement LA1500021).Upon their arrival, the rats were transferred to an animal facility, submitted to a regular 12:12h light/dark cycle. Food and water were provided ad libitum. After reception, the rats were distributed in 12 experimental groups of 5 animals each (n = 5). The different doses of TiO_2_ were administered to experimental animals by intraperitoneal injection as detail in a previous study [22]. Four different doses of TiO_2_ nanoparticles were tested, respectively, 0.5; 1; 4; and 16g/kg body weight and, for each dose, the animals were euthanized after 3 different time intervals, respectively, 4 days, 1 month, and 2 months after the end of the treatment. Three control groups (n=5) received a saline injection and were sacrificed, respectively, after 4 days, 1 month, and 2 months following the same schedule. All animals were euthanized by an intraperitoneal injection of Nembutal (Pentobarbital, 60 mg/ml). Before the complete arrest of the heart, blood was collected in the cava vein [22, 23]. After 30 minutes of coagulation, blood was centrifuged 15 minutes at 3000g to harvest the serum.

Just after euthanasia, kidneys and liver were quickly fixed by immersion in Bouin Alcohol for 2 days. Fixed tissue blocs were dehydrated in graded ethanol and in butanol and embedded in Paraplast Plus® paraffin according to a standard procedure. Paraffin sections of 5 *μ*m thickness were cut on a Microm® HM 360 microtome and mounted on silane-coated glass slides. Histopathological examination of kidneys was performed after staining with periodic acid-Schiff (PAS), hemalun, and luxol fast blue [24]. Liver slices were stained with Masson's trichrome.

### 2.3. Transmission Electron Microscopy

Ultrastructural characterization of cytological alterations was made following a procedure detailed in previous publications [25, 26]. Briefly, liver or kidney tissue blocks (max. 1mm^3^) were immediately dissected after euthanasia and were kept for 2h at room temperature in fixative (2% glutaraldehyde and cacodylate buffer 0.08 M; pH: 7.4) and thereafter postfixed in 1% OsO_4_ in the same buffer. After dehydration, in graded ethanol, the blocks were finally embedded in Spurr resin (Spurr Low Viscosity Embedding Kit; Sigma-Aldrich®). Semithin sections (1*μ*m thick) were cut with glass knife on a LKB III ultramicrotome and stained with toluidine blue. For TEM, ultrathin sections were cut on a Leica® Ultracut UCT Ultramicrotome equipped with a diamond knife. The ultrathin sections were stained with uranyl acetate and lead citrate and examined in a Zeiss® LEO 906E Electron Microscope at 80kV.

### 2.4. Immunohistochemistry

Immunohistochemical method followed a protocol detailed in previous publications [22, 27]. Briefly, prior to immunostaining dewaxed sections were rehydrated and rinsed in distilled water. Specific antigens present in the tissue were unmasked by microwave pretreatment in 0.01M citrate buffer (pH:6.2) 2x 5 min. at 900W. After rinsing, endogenous peroxidase activity was quenched by a 5 min exposure to 0.5% H_2_O_2_. Before applying the primary antibodies, the pretreated sections were rinsed in PBS (0.04 M Na_2_HPO_4_, 0.01 M KH_2_PO_4_, and 0.12 M NaCl, pH 7.4) and incubated in a solution of 0.5% casein in order to bloc aspecific antigenic sites. Tissue sections were incubated overnight at 4°C with primary antibodies [polyclonal (rabbit) anti-4-Hydroxynonenal (Abcam, Cambridge, UK)] diluted at 1:75 in PBS. After rinsing in PBS, slices were treated with the anti-rabbit/peroxidase soluble complex (ImmPress™ Reagent Kit; Vector, Burlingame, CA) for 30 min at room temperature. Bound peroxidase activity was visualized by precipitation of 3,3'-diaminobenzidine 0.02% in PBS containing 0.01% H_2_O_2_. Preparation was counterstained with hemalum and luxol fast blue, dehydrated and mounted with a permanent medium. The specificity of immunolabeling was ascertained on the basis of several criteria. In each case negative controls were essayed by omitting the first or the secondary antibody or by the substitution of nonimmune serum for the primary antibody. No staining was observed on these sections under such conditions. This procedure was in accordance with the immunocytochemical procedure and negative controls detailed in a previous publication [22].

### 2.5. Morphometric Analysis

The percentage of surface occupied by cells displaying an oxidative stress was evaluated on liver sections by immunohistochemistry using an anti-4 hydroxynonenal (4-HNE) antibody. For each liver slide, the area occupied by (4-HNE) positive cells was quantified by morphometric analysis at 400x magnification. The procedure utilized software designed for morphometry and colour analysis (KS 400 Imaging system, Carl Zeiss Vision GmbH, München, Germany) [22]. This computer-assisted morphometric system was able to analyse immunocytochemical staining on the basis of difference of colour and contrast. For each histological section of liver, 5 microscopic fields were picked at random representing a total scanned area of 1.8 mm^2^. Results were expressed as mean percentage of immunoreactive area ± SD and the values were presented in Table 2.

### 2.6. Inductively Coupled Plasma Atomic Emission Spectroscopy (ICP-AES)

All kidney and liver samples (500 and 600 mg, respectively) were stored at -80°C prior to the analysis. To digest the tissue, each sample was added in a mixture of 2 ml H_2_SO_4_ 96%, 2 ml HNO_3_ 65%, and 200 mg NH_4_Cl and placed in nylon containers in a microwave oven. A digestion program of 5 minutes at 250W, 5 minutes at 400W, 5 minutes at 650W, and 5 minutes at 250W was performed. After complete cooling, solutions were transferred in a graduated matra of 10 ml. Distilled water was added to reach 10 ml. Each solution was diluted 5x and placed in the freezer at 5°C until their analysis with ICP-AES. The calibration of the analysis method was performed using Titanium Standard for ICP of 1000 ppm (1000 mg/l) (Fluka analytical, USA). 6 standards of 0.05, 0.1, 0.5, 1, 5, and 50 ppm were analysed by the spectrometer (ICP-AES Jobin Yvon, JY 38 PLUS, HORIBA Jobin Yvon) [28]. After obtaining the calibration curve, the spectrometer was calibrated again before the analysis of samples. To check the reproducibility of the data, 3 measures were realized successively on the same sample.

### 2.7. Blood Analyses

The analysis of serum samples was performed with a clinical procedure using reagent strips kit such as Kidney-2 (Arkray, NL) testing creatinine, albumin, total protein, uric acid, blood urea nitrogen, and STAT-1 (Arkray, NL) testing lactate dehydrogenase, blood urea, nitrogen, total bilirubin, alanine aminotransferase (ALT), aspartate aminotransferase (AST), and creatinine phosphokinase to highlight some markers of liver and kidney injury. Before each series of assays the analyser was calibrated and a calibration curve was established from standards provided by the firm (Arkray, NL). Reagent strips consist of a multilayer test area containing reagents that will react with the required enzymes. The colour intensity of reaction product was measured by reflection spectrophotometry using the SPOTCHEM analyser (A MENARINI SPOTCHEM ™ EZ SP-4430) as detailed in a previous publication [29].

### 2.8. Metabonomic Analyses

*(a) Urine Collect and* ^*1*^ *H NMR Analysis*. 24h before euthanasia the rats were housed in metabolism cages to collect urine samples in refrigerated tubes containing 1 ml of sodium azide 1%.

500 *μ*l of urine samples were mixed with 250 *μ*l of phosphate buffer (0.2 M Na_2_HPO_4_/0.04 M NaH_2_PO_4_, pH 7.4) prepared in a mixture H_2_O (80%)/D_2_O (20%) to minimize pH variation. Then the samples were centrifuged at 13,000 g for 10 min. 650 *μ*l of supernatants were transferred into Eppendorf tubes, in which 50 *μ*l of a 14 mM 3-(Trimethylsilyl) propionic-2,2,3,3-d4 acid (TSP) solution prepared in 100% deuterium oxide was added.

700 *μ*l of samples were transferred into a 5-mm NMR tube and ^1^H-NMR spectrum was recorded using a Bruker NMR spectrometer 500 MHz (11.8 T).

One-dimensional spectrum was acquired using a NOESYPRESAT pulse sequence (64 scans). The free induction decay (FID) was Fourier transformed and a line broadening of 0.3 Hz was applied. The spectra were automatically phase and baseline corrected using MestReNova 5.2.0 software (Mestrelab Research, Santiago de Compostela, Spain). Spectra were calibrated against d4-TSP whose reference was arbitrarily placed at 0.00 ppm.

*(b) Multivariate Analysis*. For multivariate analysis, spectrum over the range 0.08 to 10.0 ppm was divided into subregions of equal width (0.04 ppm) and the area under the curve of each of the 248 spectral subregions was integrated and converted to ASCII format. Output ASCII data were next exported to Microsoft Excel (Microsoft Office®). The regions corresponding to residual water and urea resonances (4,50 to 5,00; 5,50 to 6,00 respectively) were excluded from the analysis to suppress the residual water signal and to remove the urea signal that undergoes annoying diurnal variations. Each integrated subregion was then normalized to the total spectrum area. The resulting dataset was then exported to SIMCA+ 12.0 software (Umetrics AB, Sweden) for multivariate data analysis. Principal component analysis (PCA) supplies two types of graphs which are a simplified overview of a complex set of data. In the “scores plot”, each point corresponds to one observation, meaning the NMR spectrum of a sample. This simplified 2-D view of the dataset allows a quick identification of potential gathering or separation between groups. The second plot is called a “loadings plot” and highlights what variables (metabolites) are responsible for the discriminations between groups (each point corresponds to the mean chemical shift of a particular spectral subregion of 0.04 ppm width and consequently to the corresponding urine metabolites). These variables are finally annotated from a database to identify the corresponding metabolites. After mean-centering of the data without scaling, multivariate analysis was conducted. Principal component analysis (PCA) and partial least square discriminant analysis (PLS-DA) were carried out to discriminate the metabolic patterns between treated rats and controls as detailed in a previous publication [29].

### 2.9. Statistical Analysis

For analysis of blood parameters, the statistical comparison of data between treated rats versus controls was performed by unipaired two-tailed Student's t-test (significance level was fixed at p≤0.05). Comparison of more than two groups (for ICP-AEC values) was achieved by analysis of variance (ANOVA) followed by Dunnett's post hoc test. The probability for significance was arbitrarily set at 0.05 (*∗*p≤ 0.05) or 0.01 (*∗∗*p≤0.01).

## 3. Results

### 3.1. Characterization of NPs

**Figure 1:**
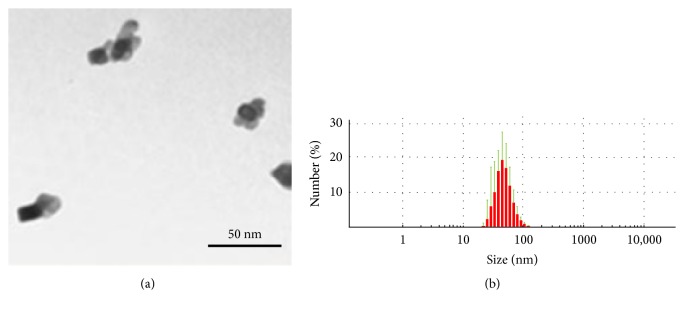
(a) Characterization (size and shape) of TiO_2_ NPs aggregates performed by transmission electron microscopy (TEM). The mean size of aggregates was calculated by morphometric analysis performed on 20 microscopic fields picked at random (n ≥ 100 aggregates). Mean nanoparticle size evaluated by this method was 34 ± 9 nm. (b) The DLS analysis indicated that the mode and dispersion around the mode of nanoparticles was 52 ± 15 nm.

The average aggregate size of TiO_2_ NPs was analysed both by electron microscopy and by dynamic light scattering (DLS) and data were summarized in Table 1. TiO_2_ nanoparticles displayed spherical shapes and showed low levels of agglomeration (Figure 1(a)). The mean size of NPs aggregates determined by DLS was 52 ± 15 nm (Figure 1(b)) and the mean size obtained by morphometrical analysis of TEM pictures was 34 ± 9 nm (Table 1). The zeta potential of the TiO_2_ nanoparticles is about – 20 mV (at pH = 7). X-ray photoelectron spectroscopy (XPS) measurements confirm that there is only titanium oxide (TiO_2_) and no traces of metallic titanium.

### 3.2. Dosage of TiO_2_ inside Tissue

**Figure 2:**
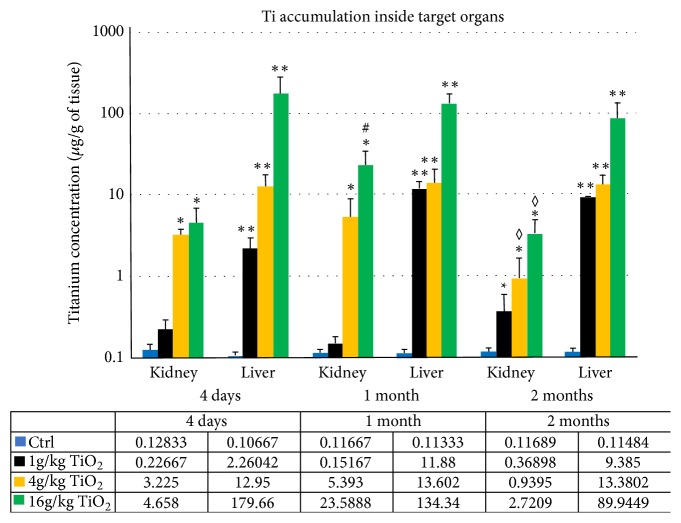
Levels of titanium (*μ*g/g of tissue) measured by inductively coupled plasma atomic emission spectroscopy (ICP-AES) inside renal and hepatic tissues from control and treated animals, sacrificed after 4 days, 1 month, and 2 months. Values are presented following a semilogarithm graph. The mean of values recorded in each group is specified in the table under the graph. Each measure was performed in triplicate. Data are mean ± SE (n=5). Significantly different from control (*∗* p≤0.05; *∗∗* p≤0.001). Significant difference 1 month versus 4 days (# p≤0.05) and 2 months versus 1 month (◊ p≤0.05).

Titanium concentrations in renal (500 mg) and liver (600 mg) tissue extracts from controls and treated animals were measured by ICP-AES. Raw data (mg/l) were converted in *μ*g of Ti/g of tissue taking into account the mass of the tissue sampled and the dilution. The distributions of Ti in kidneys and liver after 4 days, 1 month, and 2 months of exposure are illustrated in Figure 2. Titanium accumulates more efficiently in the liver than in kidneys at every time intervals with a factor 10 for the weak dose (1g/kg BW), a factor 100 for the intermediate dose (4g/kg BW), and a factor 1000 for the high dose (16g/kg BW). The accumulation in the kidneys is only significant at 4 and 16g/kg BW as compared to controls. The concentration of Ti in liver remains comparable after 1 and 2 months versus 4 days indicating that the titanium is not efficiently eliminated. In the kidney, a significant increase of titanium accumulation occurred between 4 days and 1 month in animals exposed to the highest doses. This traduced more probably a continues renal capture of circulating TiO_2_ NPs during the first weeks following administration. However, after 2 months, the renal levels of titanium decreased significantly compared to animal euthanized after 1 month revealing a progressive renal elimination of the toxic compound.

### 3.3. Histopathological Injuries

**Figure 3:**
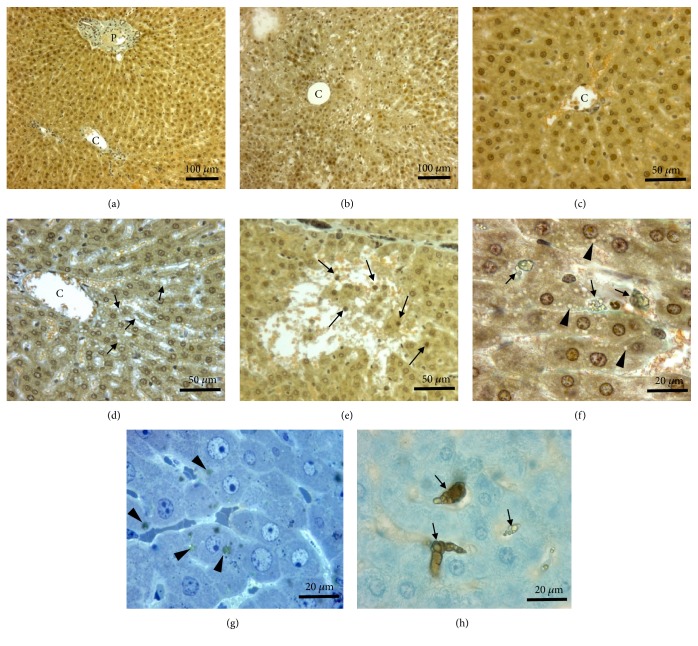
Morphology of the liver in control rats (a, c) or in animals exposed to TiO_2_ nanoparticles (16g/kg) and sacrificed after 4 days (b, d-h). (a-f) paraffin sections stained with Masson's trichrome; (g, h) semifine sections stained with toluidine blue. At low magnification, liver parenchyma of animals exposed to TiO_2_ (b) presents histopathological alterations as compared to control tissue (a). These morphological alterations were principally located around centrilobular veins (CV). In this area, numerous vacuoles (arrows) are visible in hepatocytes of treated animals (d) but not in control (c). Focally, TiO2 nanoparticles induce necrosis of hepatocyte plates (e). These necrotic areas are characterized by a lysis of liver parenchyma and the presence of numerous pyknotic nuclei (arrows) (e). At high magnification, TiO_2_ aggregates (arrows) are visible inside Kupffer cells located in the sinusoids (f, h). The TiO2 nanoparticles were internalized and aggregated inside phagolysosomes and appeared as spherical refringent inclusions (arrow). Numerous clear vacuoles (arrow heads) (f) and some spherical refringent inclusions (arrow heads) (g) are also present in hepatocytes of treated animals. (PT: portal tract).

**Figure 4:**
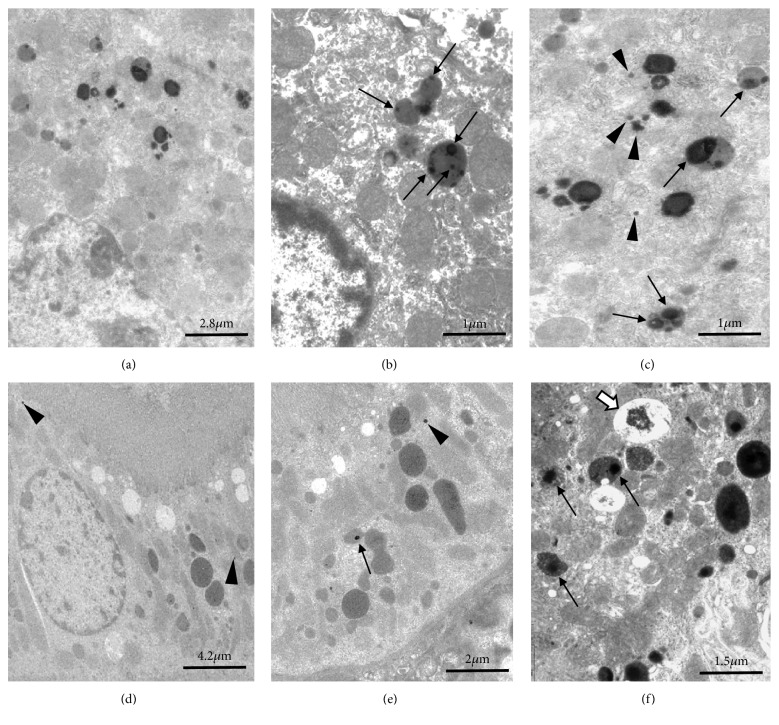
Ultrastructure of hepatocytes (a, b, c) and renal proximal tubular cells (d, e, f) of animals exposed to 1 g/Kg BW (d, e) or 16 g/Kg BW (a-c, f) and sacrificed 4 days after the end of the treatment. At low magnification (a), hepatocytes showed numerous lysosomes with heterogeneous content. At higher magnification (b, c) dense nanoparticles aggregates were observed in lysosomes (arrows) and some smaller isolated aggregates were identified free in cytoplasm (arrow heads). In kidney of animals exposed to low dose (d, e), NPs aggregates of TiO_2_ were very rare inside proximal tubular cells. Some isolated aggregates were identified focally in cytoplasm below the brush border (arrow heads) or in lysosomal compartment (arrow). At higher dose (f), nanoparticles aggregates are more frequent and increased in size. Aggregates were mainly localized in lysosomes (arrows) as in large vacuoles of endocytosis (white arrow).

In the liver, histopathological alterations are already visible at low magnification in treated animals (Figures 3(b) and 3(d)) compared to controls (Figures 3(a) and 3(c)). After intraperitoneal injection, TiO_2_ NPs reached the liver via blood circulation. NPs aggregates were internalized in Kupffer cells, probably into phagolysosomes, localized in hepatic sinusoids as well as in the periphery of portal tract (Figures 3(f) and 3(h)). These aggregates appeared as spherical refringent inclusions (Figures 3(f) and 3(h)). Some hepatocytes contained also NPs in dense cytoplasmic inclusions (Figure 3(g)). Observations at ultrastructural level confirmed the presence of dense NPs aggregates inside lysosomal compartment of hepatocytes (Figures 4(a), 4(b), and 4(c)) but also the focal presence of little clusters of TiO_2_ nanoparticles in the cytoplasm (Figure 4(c)). At high doses (4, 16g/kg BW), there was a thickening of the conjunctive capsule surrounding the liver. This hypertrophy is due to the proliferation of macrophages present in the capsule bordering the hepatic parenchyma. Around centrilobular area, hepatocytes showed a hydropic degeneration characterized by a cytoplasmic hypervacuolization (Figure 3(f)). Focally, more severe alterations were observed in liver parenchyma such as necrosis of hepatocytes located in the central area of some hepatic lobules and the formation of an edema within sinusoids. Numerous picnotic nuclei and cell fragments as well as lymphocytic infiltrates were noticed in these damaged areas of hepatic parenchyma (Figure 3(e)). These different histological and ultrastructural alterations described above in animals exposed to the highest doses (4 and 16 g / kg BW) appeared in the liver as early as 4 days after treatment. The level of alteration was chronically maintained in animals sacrificed after 1 and 2 months. Moreover, in these long-term sacrificial animals, the development of subcapsular and periportal fibrosis was observed.

**Figure 5:**
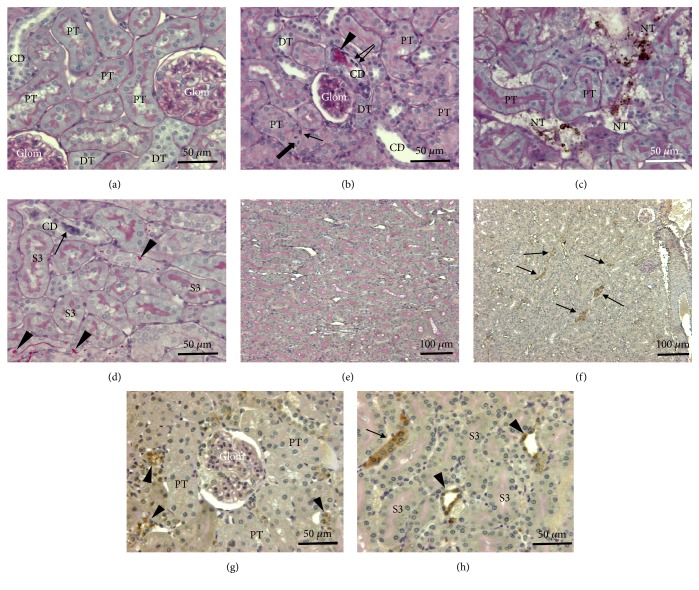
Morphology of the kidney in control rats (a, e) or in animals exposed to TiO_2_ nanoparticles (16g/kg) and sacrificed after 4 days (b-d, f-h). (a-d) Paraffin sections stained with PAS, hemalun, Luxol fast blue; (e-f) detection by immunocytochemistry of 4-hydroxynonenal (4-HNE). As compared to controls (a), kidney cortex of TiO_2_ exposed animals (b) presents generally a low level of histopathological alterations. These focal alterations are characterized by apoptotic nuclei (arrows) in proximal convoluted tubules (PT) and in collecting ducts (CD) and by the presence of PAS-positive material in the lumen of some collecting ducts (arrow head). Images of mitosis (large arrow) are frequently observed in proximal tubules. Presence of necrotic tubules (NT) characterized by a desquamation of tubular epithelium and the presence of large TiO_2_ aggregates in lumens are exceptional (c). The outer stripe of outer medulla (OSOM) (d) is also poorly impacted by the treatment. Proximal strait tubules (S3 segments) have a normal morphology. Only collecting ducts (CD) present some PAS-positive cytoplasmic inclusions (arrow heads) and exceptionally necrotic cells in the lumen (arrow). 4-HNE immunoreactivity is not present in controls (e) and is only detected in a few numbers of tubules (arrows) in treated animals (f). At high magnification (g, h) these positive tubules are identified as collecting ducts (arrow heads) and distal tubules (arrow). Glom: glomeruli; DT: distal tubules.

By contrast to the liver, kidneys were weakly impacted by the treatment. Even at high doses, no severe histological alterations were observed in kidney cortex (Figure 5(b)) or in the outer medulla (Figure 5(f)) of treated animals compared to control animals (Figures 5(a) and 5(e)). At low doses there was no change in the morphology except the focal disappearance of the brush border in some proximal tubular cells and some isolated little NPs aggregates free in cytoplasm (Figure 4(d)) or in lysosomal compartment (Figure 4(e)). Animals treated at higher doses showed some focal tubular damage that mainly affected the cortical area and to a lesser extent the medulla zone. During the acute phase (4 days), aggregates of NPs were observed in the edematous zones between cortical tubules (Figure 5(c)). Deposits of PAS-positive materials were visible in the lumen of collecting ducts indicating a glycoproteinuria (Figure 5(b)). Cellular debris also cluttered the lumen of some distal and collecting tubules (Figure 5(d)). At short and long terms, the proximal tubules seem to be the most affected. In electron microscopy, dense aggregates of nanoparticles were frequently observed in vacuoles beside brush border as also in lysosomal compartment (Figure 4(f)). Sublethal alterations such as PAS-positive intracytoplasmic inclusions (Figure 5(d)) and single cell necrosis, characterized by the presence of pyknotic nuclei within the epithelium of proximal tubules, were frequent. These different renal lesions induced by the highest doses of nanoparticles were visible in animals euthanized in the short term (4 days after the end of the treatment). The level of histological alterations decreased in animals sacrificed after 1 month and more significantly 2 months after the end of the treatment. In the long term, only very focal alterations such as brush border loss or some isolated apoptotic cells were still visible in proximal tubules of the renal cortex.

### 3.4. Immunodetection of 4-Hydroxynonenal (Marker of Oxidative Stress)

**Figure 6:**
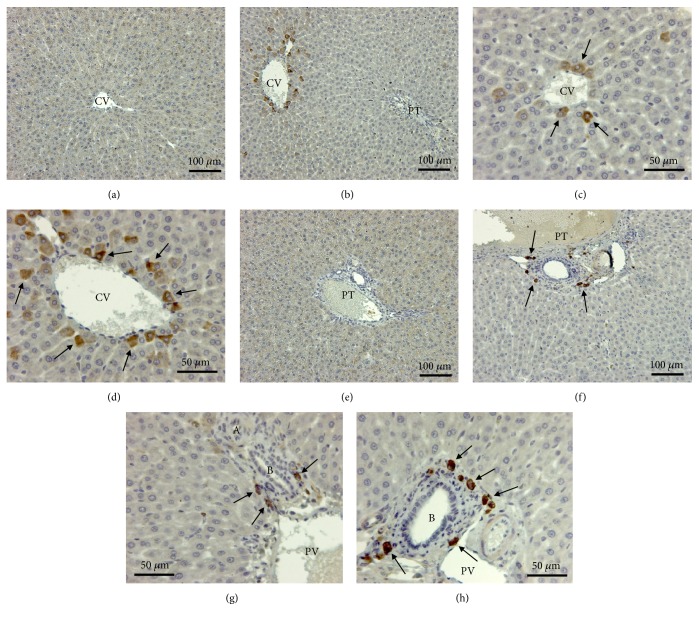
Detection by immunocytochemistry of 4-hydroxynonenal (4-HNE) in liver of controls (a, e) and in rats exposed toTiO_2_ nanoparticles (16g/kg) and sacrificed after 4 days (b-d; f-h). Oxidative stress induced a production of 4-HNE in some hepatocytes of treated rats (b) as compared to controls (a). In treated animals (c, d), hepatocytes showing an oxidative stress (arrows) were exclusively located around centrilobular veins (CV). No immunoreactivity was detected around portal triad (PT) of control rats (e) by contrast to treated animals showing some deeply stained cells (arrows) in this area (f). At high magnification, (g, h) these 4-NHE positive cells (arrows) appeared as Kupffer cells principally localized in Mall's space around bile ducts (B). PV: branches of portal vein; A: branches of hepatic artery.

The evaluation of the oxidative stress was performed by immunohistochemistry using an anti-4 hydroxynonenal antibody (4-HNE). There was no immunoreactivity in the liver of control animals as well in centrilobular area (Figure 6(a)) than around portal space (Figure 6(e)). No sign of oxidative stress was evidenced by immunohistochemical detection of 4-HNE in the liver of animals exposed to the dose of 0.5g/ kg BW and only a very few number of positive cells, identified as Kupffer cells, were present in liver of rats exposed to 1g/kg BW (Table 2). By contrast, lipid peroxidation was observed in rats exposed to two highest doses of TiO2 NPs (4 and 16 g/kg BW) and affected, respectively, 1 to 3 % of liver parenchyma (Table 2). These % remained stable along the time. In 16g/kg treated rats, the oxidative stress affecting hepatocytes presented a heterogeneous distribution inside the liver (Figures 6(b) and 6(f)). Indeed, the immunostaining was restricted to hepatocytes localized around the central vein (Figures 6(c) and 6(d)). Kupffer cells in Mall's space around bile ducts (Figures 6(g) and 6(h)) showed also a strong immunoreactivity. In the kidney, the outer stripe of outer medulla (OSOM) is the most affected area. Positive 4-HNE cells are mainly localized in distal and collecting tubules (Figures 5(f), 5(g), and 5(h)).

### 3.5. Dosage of Renal and Hepatic Enzymes

Among the essayed parameters, only aspartate aminotransferase (AST) and uric acid showed statistically significant differences between treated animals and controls regardless of the dose or the exposure time (Table 3). At 4 days and 2 months, AST in animals treated with high doses (4, 16g/kg BW) significantly increased. At 4 days the level of uric acid significantly increased only for the highest dose. By contrast, animals sacrificed after 2 months showed a significantly increase of uric acid for concentrations of 1 g/kg and 16g/kg BW.

### 3.6. Metabonomic Analyses

**Figure 7:**
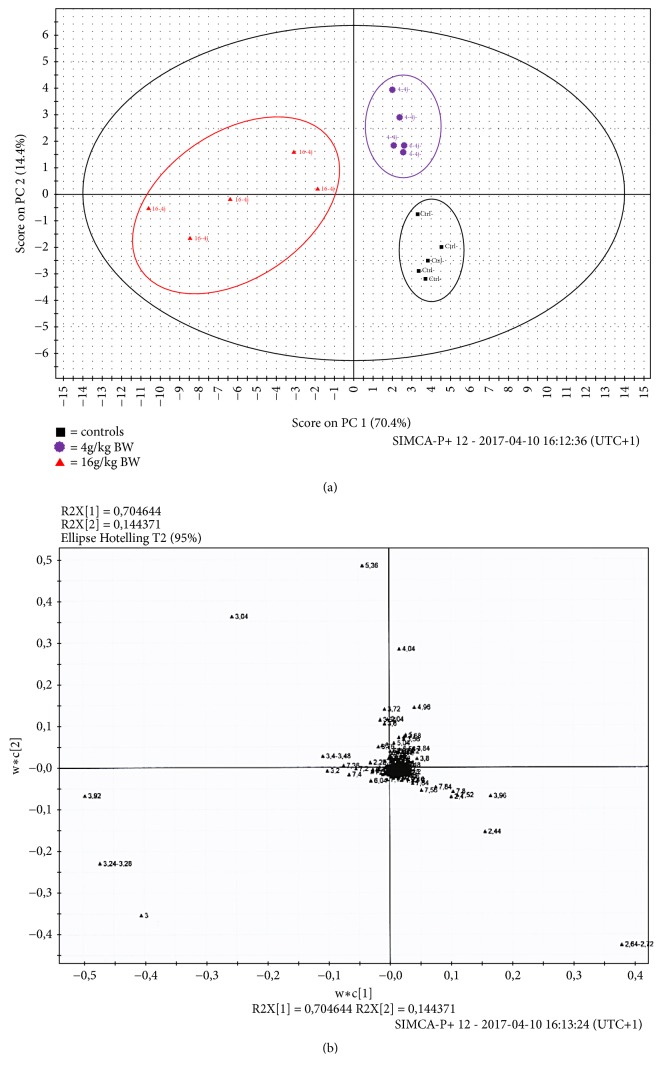
PLS-DA scores plot (a) derived from ^1^H NMR spectra of urine samples at day 4 after the intraperitoneal administration of TiO_2_ NPs at a dosage of control (black), 4g/kg BW (purple), and 16g/kg BW (red). (b) PLS-DA loadings plot corresponding to (a). A two-component PCA model was performed with a total variance of 84.8% explained (PC1 = 70.4%, PC2 = 14.4%). In the PCA score plot, 15 samples were clearly divided into three clusters marked as groups 1 (control in black), 2 (TiO_2_ 4g/Kg BW in violet), and 3 (TiO_2_ 16g/Kg BW in red), respectively. Plots given by the SIMCA-P+ software (version 12.0). The ellipse shows 95% confidence intervals.

**Figure 8:**
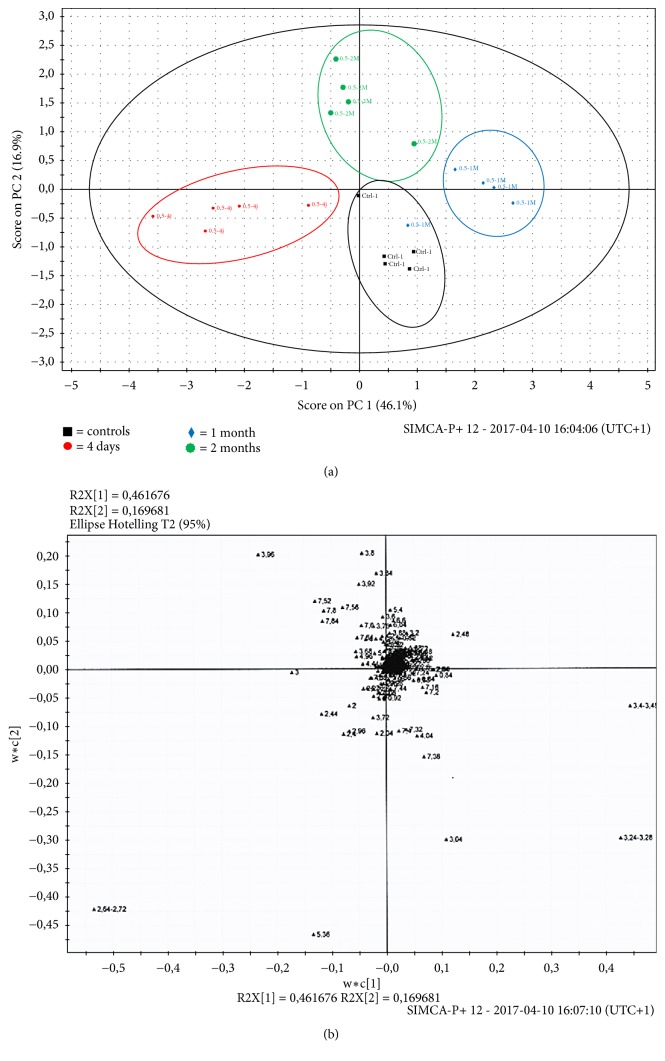
PLS-DA scores plot (a) derived from ^1^H NMR spectra of urine samples at the dose of 0.5g/kg BW according to different exposure period: control (black), 4 days (red), 1 month (blue), and 2 months (green). (b) PLS-DA loadings plot corresponding to (a). A two-component PCA model was performed with a total variance of 63% explained (PC1 = 46.1%, PC2 = 16.9%). In the PCA score plot, 20 samples were clearly divided into four clusters marked as groups 1 (control in black), 2 (TiO_2_ 4 days in red), 3 (TiO_2_ 1 month in blue), and 4 (TiO_2_ 2 months in green), respectively. Plots given by the SIMCA-P+ software (version 12.0). The ellipse shows 95% confidence intervals.

**Figure 9:**
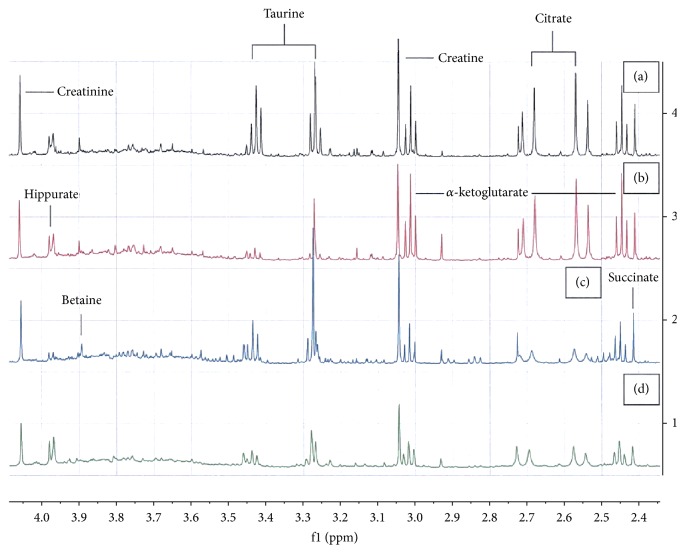
^1^H NMR spectra of urine samples (2.45–4.1) at various times of exposure ((a) control, (b) 4 days, (c) 1 month, and (d) 2 months) following the administration of TiO_2_ NPs at the dose of 0.5g/kg BW.

After intraperitoneal administration of TiO_2_ NPs, the analysis of the 1H-NMR spectra of urine collected 24h before sacrifice at day 4 showed several changes in the metabolite levels. The most important changes appeared at high doses of TiO_2_ NPs (Figure 7). Partial least squares discriminant analysis (PLS-DA) was used to analyse the metabonomic profile of urine. The scores plot (Figure 7(a)) highlighted a clear separation of control samples from those collected from animals receiving either 4 or 16 g/kg BW. For multivariate analysis, principal component analysis (PCA) revealed that the three groups were clustered separately, with 84.8% of the total variance among the three groups represented by the first two principal components (PCs), where principal component 1 (PC1) and PC2 explained 70.4% and 14.4% of the variance, respectively (Figure 7). This separation was statistically significant (p < 0.05). The changes in urine composition were identified using the loadings plot (Figure 7(b)) showing an increase in creatine, creatinine, allantoin, taurine, hippurate, and trimethylamine N-oxide (TMAO) and a decrease in citrate, *α*-ketoglutarate, transaconinate, acetate, and succinate. The summarized data of the urine metabolite levels that change in controls and treated animals were exposed in Table 4. By contrast to high doses, animal exposed to 0.5g/kg and sacrificed at day 4 presented a metabonomic profile radically different, an increase of *α*-ketoglutarate, citrate, succinate, transaconinate, and acetate and a decrease in allantoin and taurine. This metabonomic signature did not traduce a hepatic or renal impairment as it was the case with high doses. However, the fluctuations in different urinary metabolites already present at low dose suggested a precocious adaptation of the cellular metabolism in response to the oxidative stress induced by TiO_2_ NPs. A time course study of the metabonomic signature of animals exposed to 0.5 g/kg was also performed between 4 days and 2 months and presented in Figure 8. For multivariate analysis, principal component analysis (PCA) revealed that the four groups were clustered separately, with 63% of the total variance among the four groups represented by the first two principal components (PCs), where principal component 1 (PC1) and PC2 explained 46.1% and 16.9% of the variance, respectively (Figure 8). The analysis of the 1H-NMR spectra of urine of animals treated at low dose (0.5 g/kg BW) showed large variations of urinary metabolites along the time (Figures 8(a) and 8(b)). On Figure 9, 1H-NMR spectra of urine samples of animals treated with 0.5 g/kg BW at different times of exposure showed a decrease in *α*-ketoglutarate, citrate, succinate, taurine, creatinine, and betaine. This evolution of metabonomic signature of mains actors of Krebs cycle suggested a progressive degradation of energetic production along the time.

## 4. Discussion

The toxic effects associated with nanoparticles of titanium oxide in humans are mainly long-term effects resulting from chronic exposure by different routes (inhalation, ingestion, and transcutaneous passage from sun cream or internal production from titanium prostheses). The exposure of humans to TiO_2_ via different consumer products is estimated at 5 mg per person per day in occidental countries [30]. This average consumption can increase by a factor of 10 to 100 for some groups at risk who inhale large quantities of these particles at their workplace (paper bleaching, paint manufacture, etc.), or who consume large amount of products coated with these particles. Once incorporated into tissues, TiO_2_ nanoparticles are not eliminated and accumulate over time, which can lead to very high doses of several grams after several tens of years of exposure. It is very difficult to recreate such chronic exposures on rodent models that have a short lifespan of no more than two years. Thus, most animal toxicity studies carried out on these nanoparticles use different doses administered in a single time or over a relatively limited period of time.

In the present study, animals were exposed to TiO_2_ NPs at different doses (0, 0,5, 1, 4 and 16g/kg body weight) delivered in one pulse. Animal were euthanized after different time intervals (4 days, 1 and 2 months) in order to observe acute and chronic toxicological effects of TiO_2_. The range of doses used in our study was in accordance with other toxicological and pharmacokinetic studies [22, 31–34]. Morphological and physiological damage induced by a single i.p. injection of TiO_2_ NPs are very weak for doses inferior or equal to 1g/kg BW. Indeed, at these doses we observe no sign of oxidative stress by immunohistochemistry and no significant modifications of physiological markers of renal and hepatic functions. In order to observe morphological injuries, we opted for an intermediate dose of (4g/kg BW) and a high dose (16g/kg BW). The two lower doses (0.5 and 1g/kg BW) were used in order to detect precocious metabonomic changes. This sensible method allowed us to detect modifications of metabolome in absence of morphological alterations.

Despite the fact that oral administration is more relevant when assessing the risk of food additives, pharmacological parameters ADME (absorption, distribution, metabolism, and excretion) are quite often carried out with IP administration as the biochemical mode of exposure. So we have opted for this route of administration frequently used in similar toxicological studies performed on TiO_2_ NPs [22, 31, 34, 35]. Indeed, this route of administration makes it possible to control precisely the dose administered whereas when the compound is administered* per os* it is very difficult to estimate precisely what is absorbed by the intestine.

The liver is the major distribution site due to its high blood irrigation and the phagocytosis of NPs by Kupffer cells. The other major target organs are spleen, kidneys, and lungs [8, 35, 36]. The amount of TiO_2_ NPs in the mouse liver, spleen, lung, kidneys reached high levels 14 days after intraperitoneal administration [34]. After oral administration, Wang et al. [37] have also reported the accumulation of TiO_2_ NPs in the liver, kidneys, spleen, and lung. As shown in the present study, hydropic degeneration and a spotty necrosis of hepatocytes were detected in the liver of animals exposed to high doses of 4 and 16g/kg BW. These lesions were mainly concentrated around centrilobular vein. These alterations were clearly correlated with an oxidative stress localized in the same area of the liver. This area is the lowest oxygenated part of the hepatic lobule. It may be the reason why hepatocytes present around the central vein are particularly sensitive to oxidative stress induced by TiO_2_. Indeed, a positive correlation between tissue anoxia and the oxidative stress has been demonstrated in other organs [38, 39]. Kupffer cells are also largely impacted by oxidative stress. This phenomenon results more probably from the high propensity of these cells to internalize nanoparticles present in liver sinusoids around portal spaces [33].

In the kidneys, morphological alterations were characterized by a protein material accumulation in the lumen of some distal tubules and collecting ducts but also a swelling of the renal glomeruli. Oxidative stress was only evidenced in some distal tubules and collecting ducts present in renal outer medulla. This part of the nephron is also lesser oxygenated than the proximal section [40] and therefore probably more exposed to oxidative stress induced by TiO_2_.

Several studies have demonstrated the production of an oxidative stress following exposure to TiO_2_ NPs. Liang et al. [15] highlighted an oxidative stress and lipid peroxidation in the liver and kidneys. This was due to the decrease of superoxide dismutase (SOD) activity and glutathione peroxidase (GSH-PX) and to the increase of malondialdehyde (MDA) levels. Mice orally exposed to TiO_2_ NPs exhibited upregulated 8-hydroxyl deoxyguanosine (8-OHdG) level and DNA damage in the liver [41]. Besides, the ROS production increased, along with the inhibition of GSH-PX and SOD activities. Meanwhile, methane dicarboxylic aldehyde (MDA) levels were elevated in the liver and kidneys which might contribute to cell apoptosis. A recent study showed that the level of ROS in liver and kidney of mice knockout for Nrf2 (-/-) increased [42]. Nrf2 is a master regulator for the expression of multiple antioxidant genes. The loss of Nrf2 led to an increase of DNA damage and consequently an increase of risk of cancer. In response to DNA damage, the cells either initiate DNA repair mechanisms or trigger cell cycle arrest and apoptosis.

Pelclova et al. [16] showed that some biomarkers related to oxidative stress are upregulated in exhaled breath condensate collected from workers exposed to TiO_2_ NPs. This suggests that TiO_2_ NPs can induce oxidative stress in humans.

*In vivo* studies confirmed the presence of an oxidative stress in major organs such as the brain, liver, lung, kidney, and spleen after administration of TiO_2_ NPs via several routes (intragastric, inhalation, and intraperitoneal administration) [2]. This was confirmed by many* in vitro* studies. NPs can be internalized,* in vitro* and* in vivo*, by endocytosis, phagocytosis and micropinocytosis [43].

Oxidative stress plays a critical role in the occurrence of toxic effects related to exposure to TiO_2_ NPs and is responsible for cellular response such as apoptosis, perturbation of cell cycle, and inflammatory response. Nowadays, it is accepted that antioxidant molecules (Vitamin E, carnosine, and quercetin) can mitigate the toxic effects induced by the exposure to TiO_2_ NPs [44].

Several recent studies have demonstrated a direct relationship between the oxidant stress induced by toxins or metal oxide nanoparticles, the disruption of mitochondrial activity, and the induction of apoptosis [45–48]. These mechanisms were characterized by a decreased activity of superoxide dismutase (SOD), a decrease of main Krebs-cycles metabolites leading to a rapid fall of ATP production. These different studies reported also a significant increase of methane dicarboxylic aldehyde (MDA) content, a mitochondrial membrane depolarization, and an opening of mitochondrial permeability transition pore (MPTP). These mitochondrial alterations leading to Cyt c release and activation of caspases 3 and 9 are directly involved in the induction of apoptosis [45].

In this study, metabonomic method was coupled with hematology and histopathology to highlight possible toxic effect of TiO_2_ NPs. Metabonomic analyses allow to detect biochemical variations in urine precociously when the histopathological examination does not show injuries. Firstly, changes in several metabolites involved in energy metabolism such as Krebs cycle were observed. Krebs cycle allows the production of ATP in mitochondria from the energy released by the oxidation of acetyl-CoA. In the present study, the lowest doses of TiO_2_ caused a rapid increase of the major metabolites involved in the Krebs cycle, reflecting an increase of cellular metabolism in response to the moderate oxidative stress induced by these low doses. By contrast, administration of high doses of TiO_2_ caused a significant decrease of Krebs cycle metabolites. The decrease in activity of some key enzymes involved in Krebs cycle reveals dysfunction of the mitochondrial respiratory functions and that could induce organ failure such as kidney or liver [49]. Similar observations were reported by Tang and collaborators [50] in a metabonomic study of plasma samples collected from rats exposed to TiO_2_ NPs by inhalation. Acetate is an endogenous metabolite of *β*-oxidation of fatty acids in liver. Its increased level in urine can be explained by the metabolization of acetyl-CoA that is produced by the *β*-oxidation of fatty acids [51]. The concomitant increase of taurine and creatine may reflect liver lesions such as necrosis [52]. The modification of levels of betaine (osmolyte) can also be explained by alteration of liver and more particularly of Kupffer cells. Betaine is produced to maintain hepatic blood flow that can be affected by the increase of Kupffer cell volume during phagocytosis [53]. The loss of betaine in urine can be explained by a higher recruitment in the liver to warrant the integrity and the function of Kupffer cells. Creatinine derives from the degradation of creatine. A high level of urinary creatinine can be explained by muscle damage or kidney injuries such as glomerular involvement or renal ischemia. The occurrence of lipid peroxidation may impair cell integrity. TMAO in urine is associated with a loss of membrane integrity caused by the oxidative stress [54]. Hippurate is a metabolic product of benzoate. When animals are exposed to drugs, several studies have demonstrated that the increase of hippurate in urine is due by a disturbance of gut microbiome that can synthetize benzoate. TiO_2_ NPs are able to destabilize the bacterial flora in the intestine [55]. Allantoin is a product of free radical-induced oxidation of uric acid and is efficiently excreted in the urine and used as a biomarker of oxidative stress and lipid peroxidation [56]. Trigonelline is produced by the metabolism of niacin (vitamin B3) and is excreted in urine. The increase amount of trigonelline is certainly due to a defective mechanism in the niacin metabolism pathway [57].

In the present study, animals exposed to the highest doses of TiO_2_ (4 and 16g/kg BW) showed significantly different metabolic profiles from their control counterparts. Fluctuations in urine metabolites (increased creatine, creatinine, allantoin, taurine, hippurate, and trimethylamine N-oxide (TMAO) and decreased citrate, *α*-ketoglutarate, Succinate, and betaine) demonstrate both oxidative stress and hepatic and renal toxicities. Our findings are consistent with the study by Bu et al. [58] who found a very similar metabonomic signature in animals exposed by gavage at daily doses of TiO_2_ nanoparticles of 0.16, 0.4, and 1 g /kg BW/day during 2 weeks. In the short run, animals exposed to lowest dose (0.5g/kg BW) displayed neither metabonomic signs of hepatic or renal dysfunction. However, there were fluctuations in different urinary metabolites that reflect rather a rapid adaptation of the cellular metabolism. The adaptation includes a modification of enzymes activity involved in Krebs cycle reflecting a transitory increase of cell metabolism to promptly counteract the effects of moderate oxidative stress. After that animals exposed to low doses showed a low degradation of energetic production along the time as evidenced by the decrease of the major Krebs cycle metabolites. All of our observations indicate that the metabonomic approach is a relevant and sensitive method for studying the metabolic effects associated with exposure to nanomaterials.

## 5. Conclusions

**Figure 10:**
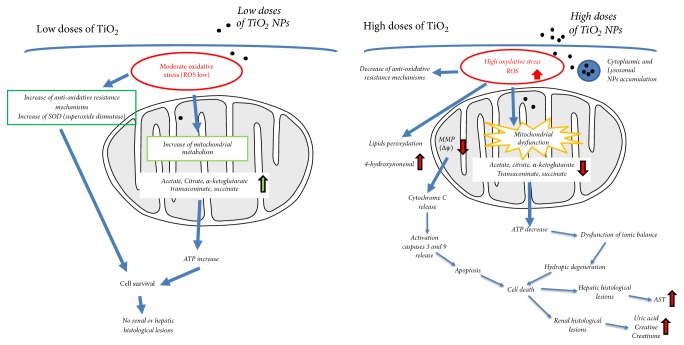
Schematic summary of the complex relationship between oxidative stress, metabolic alteration, and histological lesion induced by TiO_2_ NPs administered at low or high doses.

The present study highlighted the fact that titanium dioxide nanoparticles caused detectable histological changes only in animals treated at high doses. In the liver, lesions affecting both hepatocytes and Kupffer cells were clearly related to oxidative stress evidenced by immunodetection of 4 hydroxynonenal. In the kidney, it was the proximal tubes that were most affected by exposure to high doses of TiO_2_ NPs. These alterations were accompanied by changes in renal and hepatic function parameters that persisted chronically. By contrast, animals treated at lower doses showed no histological changes in light microscopy or significant variation of renal and hepatic function parameters. However, the very sensitive metabonomic approach allowed us to demonstrate a very early change in metabolism, even in animals exposed to the lowest doses of TiO_2_. These modifications reflected early oxidative stress induced more probably by the accumulation of TiO_2_ nanoparticles in liver and kidney cells as evidenced by ICP-AES and by observations in electron microscopy. On the basis of the results, we tried to summarize the complex relationship between oxidative stress, metabolic alteration, and histological lesion induced by TiO_2_ NPs under schematic form presented in Figure 10.

## Figures and Tables

**Table 1:** 

NPs	Mean size of aggregates (nm) determined by TEM	Mean hydrodynamic diameter (nm) determined by DLS	Z potential (mV) at pH:7
TiO_2_	34 ± 9	52 ± 15	-20.1 ± 0.5

Physical characterization of TiO2-NPs by TEM and DLS methodologies. Values indicate mean ± standard deviation (SD) for each parameter.

**Table 2:** 

	% of surface occupied by 4-HNE positive cells in liver parenchyma after 4 days	% of surface occupied by 4-HNE positive cells in liver parenchyma after 1 month	% of surface occupied by 4-HNE positive cells in liver parenchyma after 2 months
Controls	-	-	-

TiO_2_ 0.5g/kg BW	-	-	-

TiO_2_ 1g/kg BW	0.07 ± 0.03	0.05 ± 0.04	0.12 ± 0.03

TiO_2_ 4g/kg BW	0.83 ± 0.34	0.78 ± 0.28	1.02 ± 0.45

TiO_2_ 16g/kg BW	3.83 ± 1.04	4.25 ± 1.53	4.17 ± 1.67

% of area occupied by 4-hydroxynonenal (4-HNE) in liver parenchyma evaluated by morphometric analysis. Values indicate mean percentage ± standard deviation (SD).

**Table 3:** 

			Controls	0.5g/kg TiO2	1g/kg TiO2	4g/kg TiO2	16g/kg TiO2
4 days	Hepatic function	Total bilirubin (mg/dl)	0.2±0.01	0.22±0.04	0.2±0.01	0.22±0.04	0.32±0.1
		Albumin (g/dl)	3.38±0.3	3.34±0.15	3.16±0.31	3.04±0.33	2.92±0.31
		AST (IU/L)	62.6±12.4	63±12.8	84.4±21.7	101.2±26.3*∗*	120.2±20.1*∗*
		ALT (IU/L)	15.6±6	10±0.1	15.6±10.3	10±0.1	11±2.24
	Renal function	Total proteins (g/dl)	5.32±0.28	5.06±0.015	5.16±0.15	4.52±0.33	4.72±0.28
		BUN (mg/dl)	13±4.1	20.4±4.9	8.8±1.8	9.8±3.35	22.8±11.5
		Uric acid (mg/dl)	1.1±0.17	1.04±0.05	1.18±0.13	1.14±0.17	1.4±0.16*∗*
		Creatinine (mg/dl)	0.32±0.04	0.3±0.01	0.3±0.01	0.32±0.04	0.38±0.08

1 month	Hepatic function	Total bilirubin (mg/dl)	0.22±0.04	0.22±0.04	0.22±0.04	0.22±0.05	0.2±0.01
		Albumin (g/dl)	3.56±0.34	0.06±0.01	4.08±0.45	3.75±0.6	3.4±0.7
		AST (IU/L)	68±20.6	64.5±13.1	62.6±26.3	58.75±15.8	97±35.8
		ALT (IU/L)	14±3.8	13.5±0.3	13.4±3.2	12±4	19±7.8
	Renal function	Total proteins (g/dl)	5.84±0.7	4.36±0.21	6.3±0.63	5.75±0.8	5.43±0.9
		BUN (mg/dl)	20.2±4.1	20.5±3.8	15.8±1.9	18±2.6	19.66±3.05
		Uric acid (mg/dl)	1.34±0.19	1.33±0.08	1.02±0.04	1.1±0.14	1.26±0.12
		Creatinine (mg/dl)	0.46±0.1	0.43±0.11	0.42±0.13	0.4±0.2	0.5±0.1

2 months	Hepatic function	Total bilirubin (mg/dl)	0.2±0.01	0.2±0.01	0.2±0.01	0.23±0.01	0.2±0.01
		Albumin (g/dl)	3.36±0.08	3.3±0	3.26±0.15	3.42±0.1	3.39±0.1
		AST (IU/L)	46.66±9.01	64±8.8	65.4±14.1	88.6±19.3*∗*	81.75±15.6*∗*
		ALT (IU/L)	17±2	12.4±1.9	18.8±7	16.2±5.7	15±2.1
	Renal function	Total proteins (g/dl)	6.03±0.31	5.6±0.04	5.7±0.2	5.35±0.08	5.6±0.1
		BUN (mg/dl)	20±3.33	22.8±2.24	21.8±0.64	16.5±1.65	19.5±2.3
		Uric acid (mg/dl)	1.06±0.08	1.18±0.1	1.5±0.2*∗*	1.23±0.1	1.67±0.14*∗*
		Creatinine (mg/dl)	0.43±0.08	0.5±0	0.46±0.07	0.48±0.08	0.55±0.07

Summary table of average renal and hepatic enzyme levels (as mean ± SD) according to different exposure times to TiO_2_ NPs. Significantly different from control (*∗* p ≤ 0.05).

**Table 4:** 

Metabolites	Chemical shift	TiO_2_ 0.5g/kg BW	TiO_2_ 4g/kg BW	TiO_2_ 16g/kg BW
Allantoin	5.38	*↘*	*↗*	*↗*

Creatinine	3.05	→	*↗*	*↗*

Creatine	3.93, 3.04	→	*↗*	*↗*

Taurine	3.44, 3.40	*↘*	*↗*	*↗*

TMAO	3.28	→	*↗*	*↗*

Hippurate	7.84, 7.64, 7.56, 3.96	→	*↗*	*↗*

*α*-ketoglutarate	3.44, 3.00	*↗*	*↘*	*↘*

Citrate	2.64, 2.56	*↗*	*↘*	*↘*

Betaine	3.27	*↘*	→	→

Trigonelline	9.1, 8.8, 8.1, 4.4	*↗*	→	→

Succinate	2.43	*↗*	*↘*	*↘*

Transaconitate	6.66	*↗*	*↘*	*↘*

Acetate	1.95	*↗*	*↘*	*↘*

Summary of the variations of urine metabolites induced by intraperitoneal administration of TiO2 NPs at day 4 after the end of the treatment. *↗*: increase compared to control; *↘*: decrease compared to control; →: no change.

## Data Availability

All data used to support the findings of this study are included within the article.

## Abbreviations

4-HNE: 4-Hydroxynonenal
4-HHE: 4-Hydroxyhexenal
8-OHdG: 8-Hydroxyl deoxyguanosine
ALT: Alanine aminotransferase
AST: Aspartate aminotransferase
COX-2: Cyclooxygenase-2
DAB: 3.3'-Diaminobenzidine
DLS: Dynamic light scattering
GCLC: Glutamate cysteine ligase
GSH: Reduced glutathione
GSH-PX: Glutathione peroxidase
GSR: Glutathione reductase
GSSG: Oxidised glutathione
HO-1: Heme-oxygenase-1
ICP-AES: Inductively coupled plasma atomic emission spectroscopy
MAPK: Mitogen-activated protein kinase
MDA: Malondialdehyde
NADPH: Nicotinamide adenine dinucleotide phosphate
NF*κ*B: Nuclear factor kappa B
NPs: Nanoparticles
Nrf2: Nuclear factor erythroid 2-related factor 2
PAS: Periodic acid-Schiff
PCA: Principal component analysis
PLS-DA: Partial least square discriminant analysis
ROS: Reactive oxygen species
SOD: Superoxide dismutase
TEM: Transmission electronic microscopy
TLR4: Toll-like receptor 4
TMAO: Trimethylamine N-oxide
TSP: 3-(Trimethylsilyl) propionic-2,2,3,3-d4 acid.
